# Urine neutrophil gelatinase-associated lipocalin (NGAL) as a biomarker for acute canine kidney injury

**DOI:** 10.1186/1746-6148-8-248

**Published:** 2012-12-28

**Authors:** Ya-Jane Lee, Yu-Yen Hu, Yi-Shan Lin, Chun-Ting Chang, Fong-Yuan Lin, Min-Liang Wong, Hsu Kuo-Hsuan, Wei-Li Hsu

**Affiliations:** 1 Department of Veterinary Medicine, College of Veterinary Medicine, National Chung-Hsing University, Taichung, Taiwan; 2 Veterinary Medical Teaching Hospital, College of Veterinary Medicine, National Chung-Hsing University, Taichung, Taiwan; 3 Division of Chest Medicine, Department of Internal Medicine, Taichung Veterans General Hospital, Taichung, Taiwan; 4 Graduate Institute of Microbiology and Public Health, College of Veterinary Medicine, National Chung-Hsing University, Taichung, Taiwan

**Keywords:** NGAL, Kidney injury, Biomarker, ELISA, Polyclonal antibody

## Abstract

**Background:**

Biomarkers for the early prediction of canine acute kidney injury (AKI) are clinically important. Recently, neutrophil gelatinase-associated lipocalin (NGAL) was found to be a sensitive biomarker for the prediction of human AKI at a very early stage and the development of AKI after surgery. However, NGAL has not yet been studied with respect to dog kidney diseases. The application of NGAL canine AKI was investigated in this study.

**Results:**

The canine NGAL gene was successfully cloned and expressed. Polyclonal antibodies against canine NGAL were generated and used to develop an ELISA for measuring NGAL protein in serum and urine samples that were collected from 39 dogs at different time points after surgery.

AKI was defined by the standard method, namely a serum creatinine increase of greater than or equal to 26.5 μmol/L from baseline within 48 h. At 12 h after surgery, compared to the group without AKI (12 dogs), the NGAL level in the urine of seven dogs with AKI was significantly increased (median 178.4 pg/mL vs. 88.0 pg/mL), and this difference was sustained to 72 h.

**Conclusion:**

As the increase in NGAL occurred much earlier than the increase in serum creatinine, urine NGAL seems to be able to serve as a sensitive and specific biomarker for the prediction of AKI in dogs.

## Background

Acute kidney injury (AKI) shows high mortality and occurs in patients undergoing both cardiac or non-cardiac surgery [1], and among those in an intensive care unit (ICU). However, due to the lack of a consensus definition of AKI severity, the early recognition of AKI has been difficult. In the past, the Acute Dialysis Quality Initiative (ADQI) group published RIFLE criteria in which the severity of AKI was graded into Risk, Injury and Failure based on changes in serum creatinine and urine output; using this approach chronic renal failure was classified into the Loss and End stage [2]. More recently, the Acute Kidney Injury Network (AKIN) modified these criteria to increase the sensitivity, and recommended that a small change in serum creatinine (more than 26.5 μmol/L from baseline) within 48 h be recognized as stage I AKI [3]. Despite the lack of gold standard criteria for AKI in veterinary medicine, the application of the human criteria has been verified as applicable to dogs with AKI [4,5].

Based on the consensus definition, the prediction of AKI has been improved. At present, AKI is mainly diagnosed based on an elevation in serum creatinine; however, serum creatinine can be affected by age, gender, muscle mass, and hydration status; furthermore, it only rises after a loss of renal function greater than 50% [2]. In addition, the increase in serum creatinine cannot be a real time indicator of renal injury since days are needed to reach a steady state between the production of serum creatinine and the decrease in excretion of serum creatinine [6,7]. As AKI is important not only in humans but also in veterinary medicine, other biomarkers that can be used to indicate AKI at early state are needed.

Neutrophil gelatinase-associated lipocalin (NGAL, also called lipocalin 2 or 24p3), a small 25 kDa protein that belongs to lopocalin family, is highly expressed during ischemic renal injury in animal models [8]. Very recently, a number of studies have indicated that serum and urine NGAL are sensitive and specific biomarkers for the prediction of AKI [9-11] as well as the development of AKI among human patients after an operation, such as cardiac surgery [12-15], non-cardiac surgery [16], renal transplantation [17,18], and among trauma patients [19], as well as when there is IgA nephropathy [20].

Nevertheless, the role of NGAL in dogs has never been studied. Therefore the aim of the present study was to determine whether NGAL can be used for the early prediction of AKI in dogs. In this work, we first established an ELISA that allows the measurement of canine NGAL. Additionally, the validity of serum NGAL and urine NGAL for the early identification of AKI in post surgery dogs was determined.

## Methods

### Amplification of NGAL cDNA

Mammary gland tumors (MGT) have been reported to highly express NGAL [21] and therefore, in order to clone the NGAL gene sequence, total RNA was extracted from MGT by reagent TRIzol (Invitrogen) according to the manufacturer’s instructions. The first-strand cDNA was synthesized by SuperScript III reverse transcriptase (Invitrogen) using RNA (1 μg) and primers (50 μM). Subsequently, NGAL was amplified by polymerase chain reaction (PCR) with primer designed according to GenBank accession no. XM_548441 (NGAL-F: 5^′^-ATGACCCAAGTTCTCCTGTG; NGAL-R: 5^′^-TCACTCATCAATGCACTGGTC). The thermocycling conditions started with 94°C for 5 min followed by 35 cycles of heat denaturation at 94°C for 30 s, primer annealing at 60°C for 30 s, DNA extension at 72°C for 30 s and a final extension at 72°C for 5 min. The resulting PCR product with predicted size was isolated and cloned into PCR 2.1 TOPO vector (Invitrogen), designated c-NGAL/TOPO. The identity of the NGAL was verified by automated sequencing (Mission Biotech, Taipei, Taiwan).

### Construction of a plasmid expressing recombinant NGAL

Initially, the canine NGAL gene was amplified by PCR from the c-NGAL/TOPO plasmid using the primer set F-*Bam*H: 5'-aggatccaatgacccaagttctcctg-3' and R- *Xho*: 5’-ttctcgagctcatcaatgcactggtc-3‘. The PCR conditions were similar to that used for NGAL amplification from tissue, except for primer annealing at 49°C. Subsequently, the PCR product was digested with *Bam*H I/*Xho* I and cloned into pET32b. The resulting plasmid was confirmed by bi-directional sequencing.

### Preparation of recombinant dog NGAL

Protein expression was carried out in *E. coli.* strain BL21 AI (Invitrogen) following the procedures described previously [22]. Briefly, protein expression was induced with isopropyl-β-D-1-thiogalactopyranoside (IPTG, 0.8 mM) and 0.2% L-arabinose (CALBIOCHEM) at 16°C for 24 h. The bacteria pellets were resuspended in lysis buffer (500 mM NaCl, 500 mM Tris–HCl, 20 mM imidazole, and 8 M urea; pH 7.4) and this was followed by three sonication cycles of 30 sec each (550 Sonic Dismembrator; Fisher Scientific). After a second centrifugation, the cell supernatant containing the recombinant proteins was recovered and purified using chelating Sepharose Fast Flow (GE Healthcare) by following the manufacturer’s instructions. Finally, the bound protein was eluted in 4 mL elution buffer (0.05 M Tris–HCl, 0.5 M NaCl, 400 mM imidazole, and 8 M urea, pH 7.4) and then dialyzed against 1x PBS at 4°C to remove excess imidazole and urea.

### Western blotting

Purified proteins were separated by 12% sodium dodecyl sulfate polyacrylamide gel electrophoresis (SDS-PAGE) and then electrophoretically transferred to nitrocellulose membrane. The procedures for Western blot analysis followed those in a previous report [23]. Briefly, after a blocking step in PBS containing 0.1% Tween-20 (PBST) and 5% fat-free dried milk for 1 h at room temperature, the membranes were incubated with 1:5000 diluted anti-His tag antibody (AbD Serotec) or 1:500 diluted rabbit immunized with recombinant NGAL in PBST-5% dried milk at 4°C for overnight. Next, the filter was washed in PBST, which was followed by incubation with horseradish peroxidase (HRP)-conjugated secondary antibody in PBST-2% dried milk at room temperature for 1 h. After extensive washing with PBST, the filter was developed using an enzyme-linked chemiluminescence system (ECL, Amersham, GE Healthcare) and exposed to X-ray film.

### Production of antibodies against NGAL

Three BALB/c mice, aged eight weeks and three-month old New Zealand white rabbits were immunized with 50 μg NGAL/mouse (200 μg/rabbit) mixed with complete Freund's adjuvant (Sigma). After initial immunization, two boosters of the same dose were given at two-week interval. Blood was collected from the mouse submandibular vein by a lancet and from the ear vein of the rabbits. After centrifugation, the plasma was transferred to a new tube and stored at -20°C until use. The study was approved by the Institutional Animal Care and Use Committee of National University of Chung-Hsing University, permit number: 100-66.

### Establishment of a sandwich ELISA for the detection of canine NGAL

Our NGAL ELISA was optimized as follows. A checkerboard titration was initially conducted using various concentrations of the recombinant canine NGAL protein (at concentrations of 0, 2.48, 7.4, 22.3, 67, 200, and 600 pg/mL) in combination with serially diluted rabbit serum (dilutions of 1:100, 1:200, 1:400, 1:800, 1:1600, and 1:3200). All combinations were repeated in triplicate. It was found that the 1: 800 dilution of rabbit serum was sufficient to give strong signal with the lower concentration of NGAL proteins and, based on this, 1:800 diluted rabbit serum was used as the capture antibody in our Sandwich ELISA. The dilution of the mouse serum, as the detector antibody, was optimized following the same strategy.

After optimization, the ELISA conditions were used to detect NGAL in clinical samples. To do this, a 96-well microplate was coated with 100 μL of rabbit sera containing anti-NGAL polyclonal antibody (the capture antibody) at 1:800 dilution in coating buffer (0.15 M sodium carbonate, 0.35 M sodium bicarbonate, pH 9.6) at 37°C for 1 h. After blocking, the test samples (20-fold diluted with PBS-T containing 5% dried milk), recombinant NGAL protein at known concentrations as positive controls, and calibrator (diluted with blocking buffer) were individually added to each well and incubated at 4°C overnight. After washing with PBS-T, each well received 100 μL of mouse anti-NGAL polyclonal antibody (detector antibody, 1:3000 diluted in PBS-T containing 5% fat-free dried milk). After incubation at 37°C for 1 h, HRP conjugated goat anti-mouse IgG antibody (5,000 fold diluted in blocking buffer) was added to the wells. After 1 h incubation, the result was visualized using tetramethylbenzidine (TMB) substrate kit (Clinical Science Laboratory, Inc.). The optical density (OD) of each well was read at 450 nm using a microplate reader (TECAN). Each sample was analyzed in triplicate, and the OD of the triplicates was averaged. Samples with an OD value three times greater than the negative control serum were considered positive. A commercial Dog NGAL ELISA kit (BioPorto Diagnostics, kit 043) was compared with our results to assess the performance of our sandwich ELISA. Linear regression was conducted to measure the correlation between the commercial ELISA kit and our sandwich ELISA.

### Cases and sample collection

In this study, cases that had undergone surgery at the Veterinary Teaching Hospital, National Chung Hsing University, between 2009 and 2011 were enrolled. To obviate postoperative volume depletion and to avoid prerenal azotemia, all dogs received fluid transfusion for maintenance during and after surgery. Prior to the surgery, blood and urine samples were collected, while clinico-pathological information was simultaneously recorded. In addition, demographic information, including age, sex, body weight, and surgery type of each case, was also recorded. The anesthesia protocol was individually set for each patient. Generally, the preanesthetic agents, including opioid, midazolam/ketamine and propofol, were used for induction and isoflurane was administrated for general anesthesia. Serum and urine samples were obtained for NGAL analysis before the surgery and at frequent intervals after surgery when this was possible (12 h, 24 h, 48 h and 72 h post operation). The urine and serum supernatants were stored at -80°C until the NGAL test was conducted. For comparison, serum creatinine was simultaneously tested at the same time points.

### Data categories

Dogs with an elevated serum creatinine (> 132.6 μmol/L) or relevant renal clinical signs/history such as polyuria, polydipsia, oliguria, changes in the size of the kidneys, weight loss, obstructive urinary disease and proteinuria before surgery were excluded from the AKI study. The remaining animals were included in the study, enrolled for urine NGAL detection and used to compare the data from the in-house ELISA and commercial ELISA. Corresponding to the AKIN criteria [3], the serum creatinine of each case was taken as the baseline. The cases with a serum creatinine level that became raised to 26.5 μmol/L or more from the baseline within 48 h were considered to form the postoperative ‘AKI group’. The remaining cases formed the ‘No AKI group’. In addition, the operation duration and the use of any nephrotoxic related medicine including non-steroidal anti-inflammatory drugs (NSAIDs), angiotension-convertion enzyme inhibitors (ACE-inhibitors) and chemotherapeutic medicines such as carboplatin were also recorded.

### Statistical methods

SPSS 16.0 was used for analysis. For continuous variables, normally distributed variables are represented as a mean ± S.E.M and were compared by the independent Student’s *t* test or by one way ANOVA coupled with *post hoc* analysis by the Tukey’s method. Non-normal distribution variables were compared by the Mann–Whitney U test and recorded as medians (interquartile range, IQR). The *χ*^2^ test or Fisher’s exact test were used for categorical variable comparison. Univariate analysis of the serum and urine NGAL values by logistic regression was applied to identify the variables that were possible associated with AKI in dogs post operation.

## Results

### Preparation and verification of the NGAL antibodies

**Figure 1:**
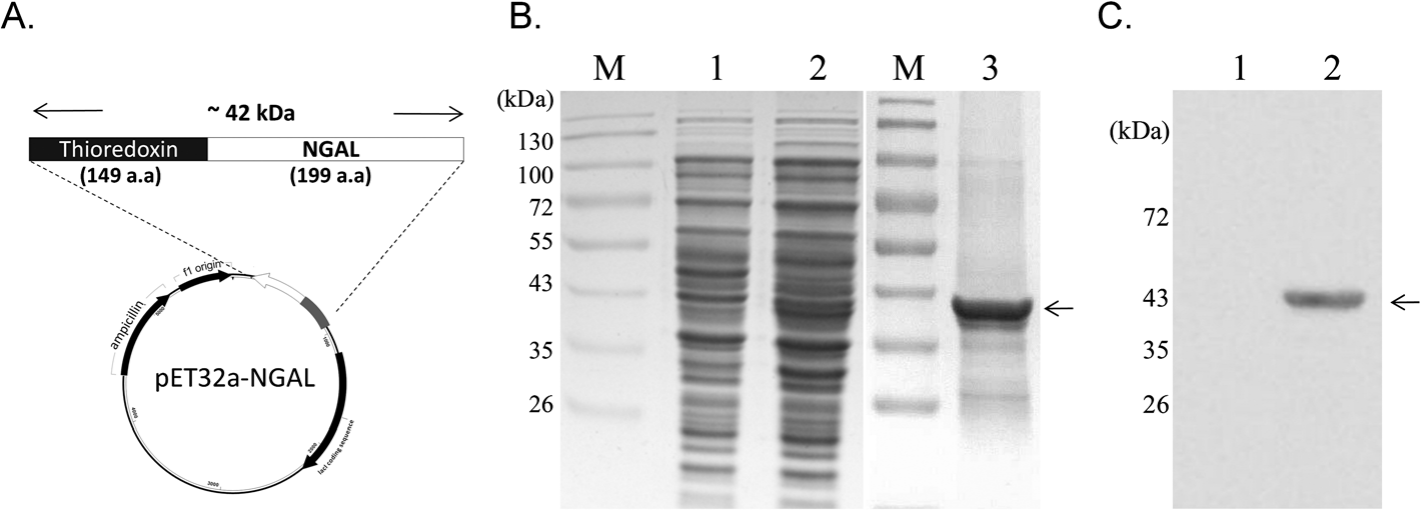
**Cloning and expression of the recombinant canine NGAL protein used to raise the antibodies.** (**A**) A schematic illustration of the NGAL construct is shown. The open reading frame of the canine NGAL was inserted into the vector pET32 downstream of the thioredoxin gene and linked to six histines. The size of recombinant canine NGAL protein was approximately 42 kDa. (**B**) The expression of canine NGAL was induced by IPTG in the host bacteria *E. coli*. As indicated by the arrowhead, recombinant NGAL, with a predicted size of approximate 42 kDa, was expressed (lane 2) and purified using Ni-NTA chromatography (lane 3). The identity of the recombinant NGAL protein (lane 2) was initially identified by Western blotting using an antibody against the His tag (**C**). M: protein size markers, lane 1: non-induction cell lysate.

To develop an in-house NGAL ELSIA, the entire coding region (597 nucleotides; 199 amino acids) of the canine NGAL gene was cloned into the expression vector pET32a (Figure 1A) and the recombinant NGAL protein was expressed and identified. As shown in Figure 1B, the thioredoxine-NGAL was successfully expressed with an expected molecular weight of approximately 42 kDa (Figure 1B). The identity of the NGAL protein was confirmed by Western blot analysis using antibody against the histidine tag (Figure 1C) and by mass spectrometry (data not shown).

**Figure 2:**
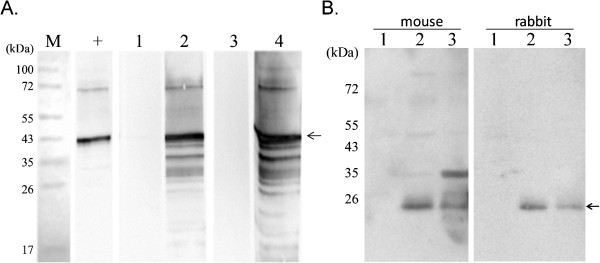
**Examination of the specificity of the two polyclonal antibodies against canine NGAL by Western blotting.** (**A**) The recombinant NGAL protein was separated by SDS-PAGE and processed for Western blot analysis. As indicated by the arrowhead, the positive control antibody (+; antibody against histidine) as well as the sera of the mice (lane 2) and the rabbit (lane 4) immunized with NGAL were able to recognize the recombinant canine NGAL protein (~42 kDa), but the pre-immunized sera of mouse (lane 1) and rabbit (lane 3) failed to detect recombinant NGAL protein. (**B**) Three clinic urine samples collected from one healthy dog (lane 1) and two renal failure dogs (lane 2 and 3) were used to examine the specificity of serum obtained from mice and rabbits immunized with recombinant NGAL. Both rabbit and mouse sera were able to recognize the endogenous NGAL (~25 kDa) as indicated by the arrowheads.

As analyzed by Western blotting, the sera from the mice and rabbits immunized with NGAL were able to recognize the recombinant NGAL protein (Figure 2A, sample 2, 4) and also specifically detected canine NGAL (25 kDa) from two renal failure dogs in which the presence of NGAL had been diagnosed by a commercial kit (Figure 2B, sample 2 and 3). This confirms the specificity of antibodies produced by the present study.

### Establishing the ELISA for NGAL detection

**Figure 3:**
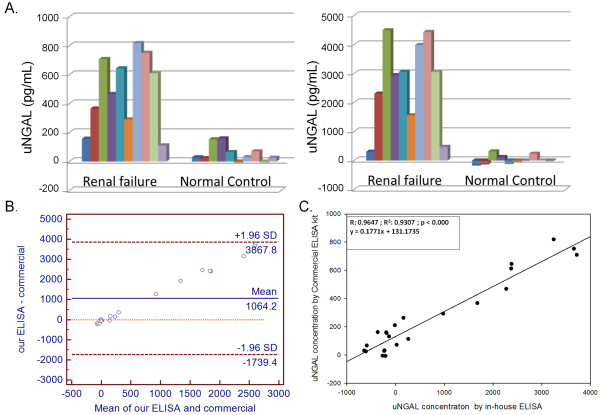
**A comparison of the results obtained by a commercial ELISA kit and by our NGAL ELISA.** (**A**) The urine NGAL(uNGAL) concentrations of twenty urine samples collected from 10 healthy and 10 renal failure dogs measured by Commercial ELISA kit (left panel) and by our ELISA (right panel). (**B**) Bland-Altman plot of uNGAL concentrations from 20 urine samples measured by the two methods. (**C**) Linear regression analysis and coefficient of correlation analysis (Software SigmaPlot 10.0) were conducted to correlate the results of these two ELISA systems. Each column represents the μNGAL concentration of a dog.

Based on the checkerboard titration result, the condition of ELISA for the detection of canine NGAL was optimized as 1:3000 diluted mouse serum and 1:800 diluted rabbit serum, which were used as the detector antibody and the capture antibody, respectively. Following optimization, twenty clinic urine samples collected from 10 healthy dogs and 10 renal failure dogs were analyzed. To evaluate the performance of the in-house NGAL ELISA, these samples at a dilution of 1:20 were also tested by a commercial NGAL kit in parallel. Results of commercial kit indicated that the OD of the urine samples from healthy dogs ranged from 0.042 - 0.521 (equivalent to 0.93 pg/mL - 159.6 pg/mL of calibrator NGAL protein), and that higher readings (0.384 - 2.396; 111.5 pg/mL - 785.6 pg/mL of the calibrator NGAL protein) were detected from renal failure dogs (Figure 3A, left panel). Consistent results were obtained using our NGAL ELISA with a higher OD being detected from the renal failure dogs (0.306 - 1.507; 306 pg/mL - 4492 pg/mL of recombinant NGAL protein) compared to the healthy group (0.114 - 0.356; 0 pg/mL - 314.3 pg/mL of recombinant NGAL protein; Figure 3A, right panel).

### Comparison of two ELISA methods

As the format, signal detection system, and assay standardization of these two ELISA systems were different in many ways. Bland-Altman plot for twenty urine samples was first performed to assess the agreement of the two methods. The mean difference between our ELISA and the commercial one is 1064.2 pg/mL, and 95% limits of agreement are -1739.4 pg/mL and 3867.8 pg/mL (Figure 3B). Linear regression and coefficient of correlation analyses were used to correlate the results of these two ELISA systems. A cross-sectional pilot study was designed to verify the standardized commercial ELISA kit against our NGAL ELISA assay. As shown in Figure 3C, NGAL concentrations in 21 urine samples and 8 calibration standards (NGAL range, 0 to 400 pg/mL) were determined by the two assays and these were found to be highly correlated (*r* = 0.96).

The equation obtained was Y = 0.1771X + 131.1735 with a coefficient of correlation 0.9646 and a *r*^2^ = 0.9307 (*p* < 0.0001); this was then used to correlate the concentration of canine NGAL determined by our ELISA assay using the purified recombinant fusion NGAL protein as calibrators with that obtained by the commercial ELISA kit using the standard calibrator NGAL protein.

### Detection of NGAL in acute kidney injury (AKI) before and after surgery using NGAL ELISA

Next, our ELISA was used to analyze the level of NGAL in dogs that had undergone surgery. Of the 39 cases investigated, 13 were for orthopedic surgery, 20 were for soft tissue surgery and six were for tumor mass removal. An increase in serum creatinine greater or equal to 26.5 μmol/L within 48 h post surgery was found to have occurred in 12 cases (30.7%), which were then classified into the AKI group. The remaining dogs were classified into the No AKI group.

The two groups were not significant different in terms of age, gender and body weight. Neither was any significant differences noted for BUN and serum creatinine between the AKI and No AKI groups prior to the surgery. However, the surgery duration of the AKI group (3.0 ± 0.8 h) was longer than that of the No AKI group (1.4 ± 0.1 h) and this *p*-value (0.05) almost reached significance (Table 1).

**Table 1:** Demographic information, laboratory information prior to the surgery and surgery duration in the AKI and non AKI groups

**Parameter**	**AKI**	**No AKI**	***P***^**a**^
Age (years)	3.7 ± 0.8, *n* = 12	4.1 ± 0.7, *n* = 26	0.749
Bodyweight (kg)	10.0 ± 2.0, *n* = 12	14 ± 2.4, *n* = 27	0.173
Admission BUN (mmol/L)	6.0 ± 0.85, *n* = 12	6.28 ± 0.43, *n* = 27	0.76
Surgery duration (h)	3.0 ± 0.8, *n* = 12	1.4 ± 0.1, *n* = 26	0.05
Admission Creatinine (μmol/L)	88.4 (53.0), *n* = 12	88.4 (26.5), *n* = 27	0.208
Gender (female)	58.3% (7/12)	70.4% (19/27)	0.486
Nephrotoxic drugs used ^b^	50.0% (6/12)	40.7% (11/27)	0.590

Continuous data agreeing with a normal distribution are presented as means ± S.E.M., otherwise, the median (IQR) are presented; categorical data are reported as a percentage.

^a^*p* < 0.05 indicates a significant difference.

^b^Nephrotoxic drugs used include NSAIDs, aminoglycoside antibiotics, ACE-inhibitors and chemotherapeutic medicine such as carboplatin.

The median urine NGAL concentrations at baseline and at different time points after surgery for each dog from the two groups are shown in Table 2. Compared with the No AKI group, the median concentration of urine NGAL in the AKI group rose significantly at 12 h (median 178.4 pg/mL vs. 88.0 pg/mL), and was also found to be higher at 24 h (median 243.6 pg/mL vs. 128.2 pg/mL *p* = 0.059), 48 h (median 201.7 pg/mL vs. 155 pg/mL *p* = 0.035), and 72 h (276.1 pg/mL vs. 70 pg/mL *p* = 0.056) (Table 2). Thus the increase in NGAL was sustained for at least three days post surgery.

**Table 2:** Median/mean of urine and serum NGAL levels as well as serum creatinine levels at various time points

**Time point**	**AKI**	**No AKI**	***p***^***a***^
Urine NGAL (pg/mL)			
0 h	51.4 (52.1), *n* = 11	83.9 (216.3), *n* = 25	0.959
12 h	178.4 (240.3), *n* = 7	88.0 (195.2), *n* = 12	0.022
24 h	243.6 (170.3), *n* = 10	128.2 (218.5), *n* = 24	0.059
48 h	201.7 (256.6), *n* = 11	155.7 (261.8), *n* = 25	0.035
72 h	276.1(269.6), *n* = 10	70.0 (257.2), *n* = 17	0.056
Max after 72 h	297.5 (193.9), *n* = 11	161.1 (199.3), *n* = 25	0.041
Serum NGAL (pg/mL)			
0 h	254.6 ± 35.5, *n* = 8	217.9 ± 22.0, *n* = 16	0.368
12 h	177.5 ± 33.8, *n* = 7	209.4 ± 27.0 *n* = 13	0.481
24 h	226.8 ± 28.9, *n* = 12	230.9 ± 21.1, *n* = 25	0.910
48 h	269.3 ± 31.1, *n* = 12	228.3 ± 23.4, *n* = 25	0.313
72 h	254.5 ± 38.1, *n* = 9	217.5 ± 22.0, *n* = 18	0.377
Max after 72 h	297.5 ± 29.2, *n* = 12	254.9 ± 22.4, *n* = 26	0.277
Serum Creatinine (μmol/L)			
0 h	79.6 ± 7.1, *n* = 12	88.4 ± 3.5, *n* = 27	0.203
12 h	77.8 ± 11.5, *n* = 6	83.1 ± 3.5, *n* = 13	0.583
24 h	104.3 ± 8.8, *n* = 12	85.7 ± 2.7, *n* = 26	0.040
48 h	103.4 ± 0.1, *n* = 12	86.6 ± 2.7, *n* = 26	0.027
72 h	113.2 ± 8.8, *n* = 10	85.7 ± 2.9, *n* = 21	0.005

Mean ± S.E.M. and median (IQR) are used to present normally and non-normally distributed continuous data, respectively.

^a^*p* < 0.05 indicates a significant difference.

In contrast, when the serum creatinine levels were examined, no distinct difference was found between the two groups of dogs until 24 h post surgery (Table 2). However, when the NGAL concentrations in the serum of the AKI and No AKI groups at the various times were compared, they were found not to be significant different between the two groups (Table 2). In addition, there was no significant difference between the AKI and No AKI groups for BUN.

Using logistic regression, it was found that the urine NGAL at 12 h and 48 h significantly increased the odds ratio for AKI. However, an increased duration of surgery also increased the odds ratio for AKI with the possibility of AKI being increased as surgery is prolonged (Table 3).

**Table 3:** **Results of the univariate analysis to identify variables significantly associated with AKI (*P*** **< 0.05) in this study**

**Variables**	**Odds ratio**	**95% Confidence interval**
Urine NGAL 12 h	1.013	1.001–1.025
Urine NGAL 48 h	1.007	1.001–1.013
Surgery duration (h)	1.969	1.018–3.811

## Discussion

In the present study, we established an antigen capture ELISA for measuring urine NGAL in dogs. Using this platform, urine NGAL was shown to act as an early predictive biomarker for acute kidney injury (AKI) after surgery. Recently, several studies have proposed that NGAL ought to be able to serve as a novel biomarker for predicting renal failure in humans [11,13,14]. However, up to the present, measurement of urine NGAL levels has not yet been included as a routine diagnosis for the prediction of renal function. Moreover, studies targeting NGAL in dogs have not been reported up to the present and as a result one might expect the detection reagent/kit to have limited availability. To be able to monitor NGAL levels in many individuals, it is important to develop a reliable and cost effective detection system.

The detection and measurement of NGAL level can be achieved by a range of methods including Western blot analysis and ELISA. So far, antibodies specific for canine NGAL are not commercially available; most of the commercial NGAL antibodies in use are specific for the human protein and cross reactivity with canine NGAL has been suggested based on gene homology and sequence similarity. Although a canine NGAL ELISA assay kit is commercially available, it is very expensive. In this context, the production of an antibody against canine NGAL is useful when developing ELISA and other assays for measuring NGAL levels in dogs. In current study, we have identified and expressed recombinant canine NGAL and used this protein to generate two different polyclonal antibodies. Compared with the results obtained from the commercial NGAL ELISA kit, we noticed that the overall NGAL concentrations of tested samples were measured as higher when our ELISA was used. The difference observed between these assays is probably, to a large degree, due to differences in the ELISA format, to higher antibody affinity, to better amplification of the detection signal, and to improved assay standardization. Nevertheless, the linear regression analysis indicated that results of in-house ELISA are highly correlated with the commercial kit, and that the NGAL concentration can be converted between these two systems using an equation. Hence, our in-house ELISA has the potential to be a useful tool when studying NGAL in dogs.

To our knowledge, this is the first study to investigate the role of NGAL in dogs with renal disease. Results of this study indicate that, as with humans, an increase in urine NGAL concentration is able to predict kidney injury in dogs after surgery. Compared with using serum creatinine for the diagnosis of AKI at 48 h post surgery, an increase in urine NGAL from the baseline is able to pinpoint AKI as early as 12 h after surgery in dogs.

Noticeably, the 2 h urine NGAL level has been found to be an effective biomarker for the prediction of AKI in several human medical studies [12-14]. In our study, the best time point for classifying dogs into those with and without AKI was much later at 12 h post surgery. As urinary catheterization of each dog after surgery cannot be accomplished, the collection of urine from dogs at 2 h post surgery is not routinely possible; therefore we may have missed the earliest point at which urine NGAL is elevated. Furthermore, due to limited clinical accessibility, urine could not be obtained from all the dogs at all time points and this resulted in missing data, which reduced the statistical power of our study. However, this preliminary study still clearly shows that urine NGAL level is able to act as a much earlier biomarker than serum creatinine when detecting post surgery AKI in dogs. Nonetheless, more research is needed to validate NGAL levels as a biomarker in dogs with various renal diseases and in dogs at different stages of disease progression.

Unlike previous human medical studies [12-14], serum NGAL was not as sensitive as urine NGAL in dogs. The reasons for this may involve the expression of NGAL occurring not only in the renal tubules but also in the respiratory tract, stomach and colon; furthermore, NGAL expression is also increased by acute bacterial infection and tissue injury [6]. Thus NGAL accumulates separately in two pools, namely a systemic and a renal pool [24]. During AKI, serum NGAL may also be derived from injured tissues other than the kidneys; these will both contribute to the systemic pool. Hence, serum NGAL concentration is not in these circumstances a specific indicator of disease with a renal origin. Additionally, studies of NGAL in human AKI have involved specific types of major surgery such as cardiac surgery [6]; however, in our study the types of surgery that the dogs underwent were diverse. The diversity of the surgery may help to obscure the serum NGAL results compared to the urine NGAL results. In contrast to serum NGAL, an increase in urine NGAL only occurs when the renal absorption ability of NGAL is disturbed by the kidney damage [25].

Despite the fact that development of post-operative AKI occurs most often after cardiac surgery in humans, it also occurs after human non-cardiac surgery [1,26]. Although the mechanism of post-surgery AKI is well understood, some risk factors such as age, emergency status, high risk surgery, ischemic heart disease [1], liver disease, body mass index and chronic obstructive pulmonary disease have been reported to predispose patients to suffer renal insult after surgery [26]. In the veterinary field, post-surgery AKI in dogs has been rarely reported, but this may be due to the lack of the early biomarker and a consensus standard. In our study, based on the AKIN criteria and the elevation of urine NGAL, AKI in dogs can now be recognized as early as 12 h post surgery.

The use of nephrotoxic medicines might have interfered with the results of this study [27]. Nevertheless, we found that the percentage of patients using nephrotoxic medicine in the AKI group was not significantly higher than in No AKI groups and thus it would seem that this variable can be neglected as an influence on our results.

Several criteria have been established to increase the sensitivity of AKI detection at the early stage; however, these criteria are based on the loss of renal function. As an applicable tool for testing renal function, serum creatinine was used as the standard for AKI. However, unlike serum creatinine, urine NGAL is a real-time indicator of active kidney damage in humans [28] and can be used to examine the progressive kidney disease. Theoretically, the use of serum creatinine levels which was a renal functional marker, even with new criteria, cannot exclude AKI. However, before a more reliable standard is established, serum creatinine is the only alternative. In this study, the elevation of NGAL was found to be a suitable biomarker that is able to detect at an earlier time point than serum creatinine in dogs with postoperative AKI. The results indicated that, like in humans, urine NGAL can be applied as a useful biomarker for the detection of AKI in dogs. However, further studies are needed in order to understand the role of NGAL in various different kidney diseases.

## Conclusions

For the first time in this study, an antigen capture ELISA was successfully established that allows the detection and quantification of canine NGAL. Urine NGAL was found to be a sensitive biomarker for AKI in dogs. In terms of the time of appearance of the two biomarkers that can be used to detect AKI, the increase in urine NGAL occurs much earlier than the increase in serum creatinine.

## Abbreviations

NGAL: Neutrophil gelatinase-associated lipocalin; AKI: Acute kidney injury; ROC: Receiver-operating characteristic curve; AKIN: Acute Kidney Injury Network; NSAIDs: Non-steroidal anti-inflammatory drugs; ACE-inhibitors: Angiotension-convertion enzyme inhibitors; IQR: Interquartile range.

## Competing interests

The authors declare that they have no competing interests.

## Authors’ contributions

YL and WH designed the experiments, analyzed the data and drafted the manuscript together. TH, YL, CC, FL, and HH performed the experiments. ML helped to draft the manuscript. All authors read and approved the final manuscript.
