# Correction: Urine neutrophil gelatinase-associated lipocalin (NGAL) as a biomarker for acute canine kidney injury

**DOI:** 10.1186/1746-6148-9-228

**Published:** 2013-11-14

**Authors:** Ya-Jane Lee, Yu-Yen Hu, Yi-Shan Lin, Chun-Ting Chang, Fong-Yuan Lin, Min-Liang Wong, Hsu Kuo-Hsuan, Wei-Li Hsu

**Affiliations:** 1

## 

After the publication of this work [1], we became aware of the fact that the values of NGAL reported in this study were concentrations of diluted clinic samples; and therefore for the actual concentration of urine NGAL, the value should multiply the dilution factor.

With exception of Figure three (Figure 1 here), all the values of urine NGAL described in this study were detected by in-house ELISA and subsequently were converted to the concentrations corresponding to the values detected with a commercial NGAL ELISA kit by the equation of Y = 0.8855X + 6.5587.

**Figure 1:**
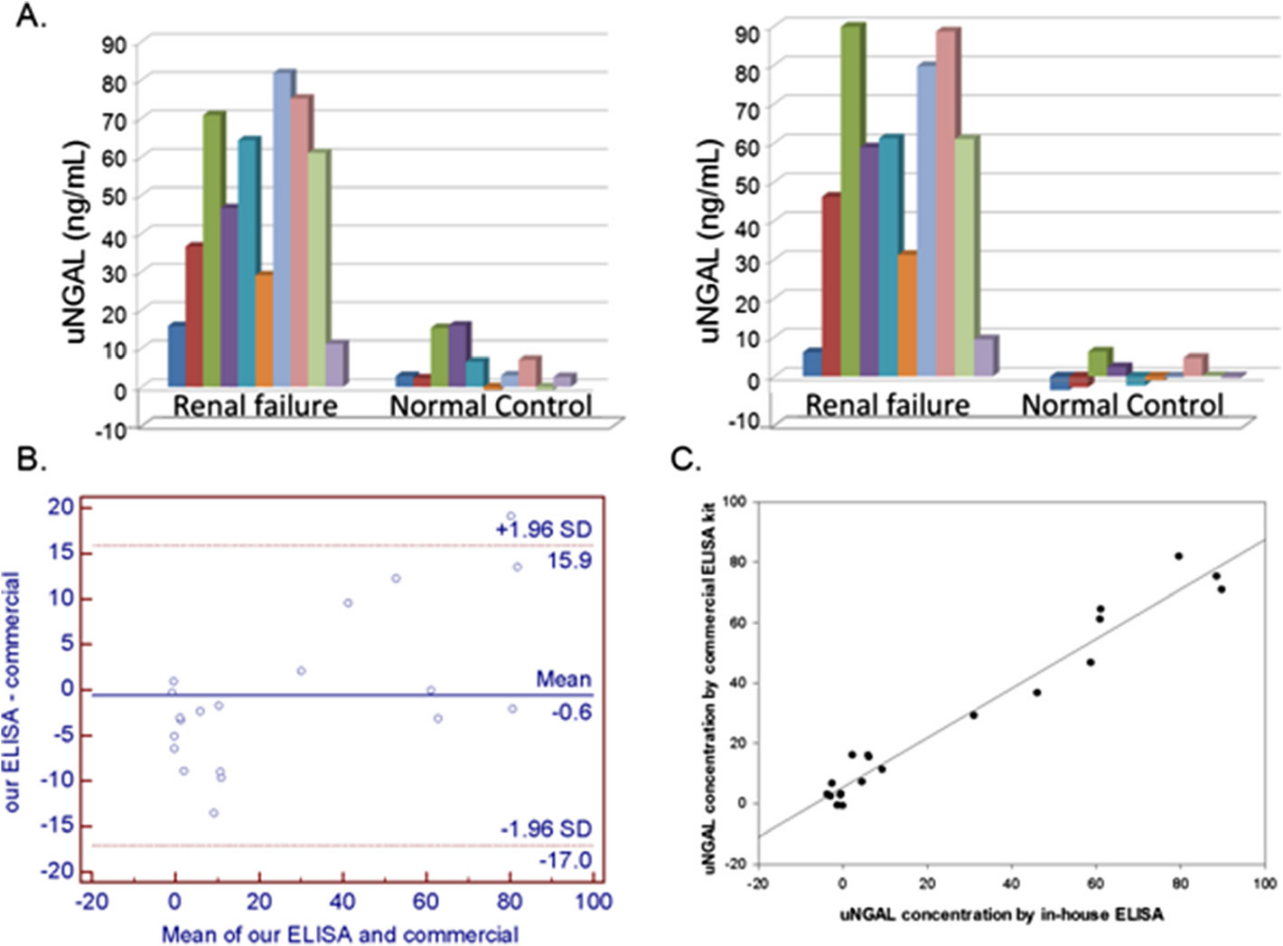
**A comparison of the results obtained by a commercial ELISA kit and by our NGAL ELISA. (A)** The urine NGAL(uNGAL) concentrations of twenty urine samples collected from 10 healthy and 10 renal failure dogs measured by Commercial ELISA kit (left panel) and by our ELISA (right panel). **(B)** Bland-Altman plot of uNGAL concentrations from 20 urine samples measured by the two methods. **(C)** Linear regression analysis and coefficient of correlation analysis (Software SigmaPlot 10.0) were conducted to correlate the results of these two ELISA systems. Each column represents the μNGAL concentration of a dog.

As a consequence, for the concentration of NGAL in urine samples listed in Table two (Table 1 here), all the values have to multiply the dilution factor: 100. And the NGAL concentrations described in abstract (subtitle of Results) should be corrected as follows: At 12 h after surgery, compared to the group without AKI (12 dogs), the NGAL level in the urine of seven dogs with AKI was significantly increased (median 17.840 ng/mL vs. 8.8 ng/mL), and this difference was sustained to 72 h.

**Table 1:** Median/mean of urine and serum NGAL levels as well as serum creatinine levels at various time points

**Time point**	**AKI**	**No AKI**	***P***^***a***^
Urine NGAL (ng/mL)			
0 h	5.14 (5.21), *n* = 11	8.39 (21.63), *n* = 25	0.959
12 h	17.84 ( 24.03), *n* = 7	8.80 (19.52), *n* = 12	0.022
24 h	24.36 ( 17.03), *n* = 10	12.82 (21.85), *n* = 24	0.059
48 h	20.17 (25.66), *n* = 11	15.57 (26.18), *n* = 25	0.035
72 h	27.61(26.96), *n* = 10	7.0 (25.72), *n* = 17	0.056
Max after 72 h	29.75 (19.39), *n* = 11	16.11 (19.93), *n* = 25	0.041
Serum NGAL (ng/mL)			
0 h	25.46 ± 3.55, *n* = 8	21.79 ± 2.20, *n* = 16	0.368
12 h	17.75 ± 3.38, *n* = 7	20.94 ± 2.70 *n* = 13	0.481
24 h	22.68 ± 2.89, *n* = 12	23.09 ± 2.11, *n* = 25	0.910
48 h	26.93 ± 3.11, *n* = 12	22.83 ± 2.34, *n* = 25	0.313
72 h	25.45 ± 3.81, *n* = 9	21.75 ± 2.20, *n* = 18	0.377
Max after 72 h	29.75 ± 2.92, *n* = 12	25.49 ± 2.24, *n* = 26	0.277
Serum Creatinine (μmol/L)			
0 h	79.6 ± 7.1, *n* = 12	88.4 ± 3.5, *n* = 27	0.203
12 h	77.8 ± 11.5, *n* = 6	83.1 ± 3.5, *n* = 13	0.583
24 h	104.3 ± 8.8, *n* = 12	85.7 ± 2.7, *n* = 26	0.040
48 h	103.4 ± 0.1, *n* = 12	86.6 ± 2.7, *n* = 26	0.027
72 h	113.2 ± 8.8, *n* = 10	85.7 ± 2.9, *n* = 21	0.005

Mean ± S.E.M. and median (IQR) are used to present normally and non-normally distributed continuous data, respectively.

^a^*P* < 0.05 indicates a significant difference.

As for Figure three (Figure 1 here), the values directly obtained by the commercial NGAL ELISA and by in-house NGAL ELISA need to multiply the dilution factors 100 or 20, respectively. We already corrected the value in the new Figure three (Figure 1 here).

We regret any inconvenience that this inaccuracy in the data used for the original analysis might have caused. We wish to thank Dr. Evence Daure for bringing this matter to our attention.
